# Radiotherapy in Pancreatic Cancer: To Whom, When, and How?

**DOI:** 10.3390/cancers15133382

**Published:** 2023-06-28

**Authors:** Michał Falco, Bartłomiej Masojć, Tadeusz Sulikowski

**Affiliations:** 1 Radiation Oncology Department, West Pomeranian Oncology Center, Strzałowska 22, 71-730 Szczecin, Poland; bmasojc@onkologia.szczecin.pl; 2 Hospicjum Św. Jana Ewnagelisty, Pokoju 77, 71-740 Szczecin, Poland; 3 Department of General, Minimally Invasive, and Gastroenterological Surgery, Pomeranian Medical University in Szczecin, 71-252 Szczecin, Poland; sulikowskit@wp.pl

**Keywords:** advanced pancreatic cancer, borderline resectable cancer, radiotherapy, radiochemotherapy, stereotactic radiotherapy

## Abstract

**Simple Summary:**

The prognosis of pancreatic cancer is always serious. In most cases, the cancer is diagnosed at an advanced stage. The treatment is based on the combination of many treatment methods. In the presented work, we focus on the analysis of the role of radiotherapy in the radical treatment of pancreatic cancer. Radiotherapy in combination with chemotherapy or as stereotactic radiotherapy after chemotherapy contributes to the improvement of the results of preoperative treatment in pancreatic cancers with borderline resectability. In locally advanced cases, the use of radiotherapy increases the probability of local cure and reduces the intensity of pain. Therefore, the use of radiotherapy in subsequent stages of treatment in patients with locally advanced pancreatic cancer should be considered.

**Abstract:**

The diagnosis rate of pancreatic cancer is steadily increasing. The average age of onset is close to 70 years. In most cases, the disease is diagnosed at an advanced stage. The indications for and techniques of radiotherapy are changing over time. The aim of this thesis is to present the role and possibilities of radiotherapy from the perspective of radiation oncologist. The most common cause of treatment failure in pancreatic cancer remains generalisation. The implementation of new systemic treatment regimens contributes to improved treatment outcomes regardless of the stage of the disease. With improved treatment outcomes in terms of the incidence of distant metastases, the impact of local curability on the length and quality of life of patients increases. Modern radiotherapy offers the opportunity to achieve high local cure rates. Postoperative radiotherapy in combination with chemotherapy seems justified in the group of postoperative pancreatic cancer patients with pT3 and pN+ features. In the group of patients with borderline resectable pancreatic cancer, the impact of radiotherapy in combination with the latest chemotherapy regimens is difficult to define clearly. In the setting of a diagnosis of advanced pancreatic cancer, radiotherapy, especially stereotactic radiotherapy, in combination with chemotherapy, contributes to improved local curability and allows to achieve a significantly reduced level of pain.

## 1. Introduction

The incidence of pancreatic cancer is steadily increasing [1]. The average age of onset is close to 70 years; diagnosis of the disease before 40 years of age is very rare [1]. In 50% of patients, distant metastases are detected at diagnosis, while regional and localised disease is diagnosed in approximately 30% and 11% of cases, respectively [2]. Overall, 60 to 70% of newly diagnosed lesions are located in the head of the pancreas, with 15% in the body and 5% in the tail [3]. Among all patients diagnosed with pancreatic cancer, the 5-year survival rate is less than 10%, while among patients with regionally advanced disease, it is approximately 13% [2].

CT with 70–85% accuracy determines the resectability of a newly diagnosed tumour. The interval from CT scan to surgery should not be more than 25 days, due to the dynamic course of the disease [4]. MRI can be helpful in localising small lesions, assessing local staging and looking for secondary lesions in the liver, peritoneum [3]. PET/CT can be helpful in detecting generalised malignancy with a sensitivity of 82% and specificity of 97% for liver lesions [5].

As with many other cancers, a variety of treatments are used to treat pancreatic cancer, including radiation. The indications for use and techniques of radiotherapy change over time. The aim of this thesis is to present the role and possibilities of radiotherapy from the perspective of radiation oncologist.

## 2. Complementary Treatment

Operative treatment is feasible in approximately 15–20% of patients with newly diagnosed pancreatic cancer. Regional recurrence rates are as high as 85% after surgical treatment alone. The unfavourable anatomical conditions, relating to the proximity of the mesenteric vessels, visceral trunk and supplying bowel, contribute to the high rate of R1 surgery in this patient group [6].

Chemotherapy based on gemcitabine or the FOLFIRINOX regimen is recommended for follow-up treatment after surgery, while the role of chemoradiotherapy is not clearly established. In the CONKO-001 trial, the use of six cycles of gemcitabine resulted in a statistically significant improvement in the 5-year disease-free survival (5yDFS) from 7% to 16.6% and 5-year overall survival (5yOS) from 10.4% to 20.7% [7]. No data are available for locoregional recurrence rates in this study. The combination of gemcitabine with capecitabine compared with gemcitabine in monotherapy increased the rate of 5yDFS from 11.9% to 20.9% and 5yOS from 16.3% to 28.8% [8]. In the aforementioned study, relapse occurred in approximately two-thirds of patients in both arms, of which locoregional relapse was diagnosed in 50% of patients and generalisation of the tumour process in 80% [8]. Recommendations for the use of the FOLFIRINOX regimen result from a comparison of the regimen with gemcitabine in a group of patients with generalised cancer. In the aforementioned group of patients, after the FOLFIRINOX regimen, the median time to recurrence was 6.4 months compared with 3.3 months in the group of patients receiving gemcitabine in monotherapy. This came at the price of significantly more frequently observed side effects [9]. The RTOG 9704 study compared the use of gemcitabine versus 5 Fu in combination with chemoradiotherapy after surgical treatment of pancreatic cancer. Chemoradiotherapy in both arms consisted of continuous 5 Fu infusion and irradiation with a dose of 50.4 Gy in 28 fractions. The study achieved a 5yOS of 22% in the gemcitabine group and 18% in the 5 Fu group [10]. Significantly fewer locoregional recurrences were observed in the chemoradiotherapy trial (35%) compared with the previously mentioned studies, despite a lower R0 resection rate in the RTOG 9704 trial (42%) [9,10]. The proportion of patients experiencing distant metastases remained similarly high (73%) [10].

A retrospective analysis of Surveillance, Epidemiology, and End Results data for patients treated between 2004 and 2016 showed a benefit of chemoradiotherapy over postoperative chemotherapy (median OS 23 m vs. 22 m), especially in patients with pT3 and pN+ features [11]. The results of a retrospective study from Italy show that in follow-up treatment with postoperative chemoradiotherapy, radiation dose is one of the most important factors. The median survival time differed significantly in favour of patients who received doses in the range of 50–55 Gy and above, compared to those who received irradiation with lower doses [12].

## 3. Neoadjuvant Treatment

In an analysis based on data from the National Cancer Database (NCDB), the results of neoadjuvant systemic treatment were compared with subsequent treatment with surgery to immediate surgery in a group of patients with stage I and II pancreatic cancer. A significantly longer median OS of 26 months was observed in the neoadjuvant treatment group compared to 21 months in the surgically treated group [13]. In a subsequent analysis from the NCDB, significantly worse treatment outcomes in the form of lower median survival were observed in the group of patients who received preoperative chemoradiotherapy alone compared with those who received chemotherapy alone or combination chemoradiotherapy with chemoradiotherapy (22.9 m vs. 25.6 m vs. 26.9 m, respectively) [14]. A retrospective analysis presented by Cloyd et al. compared groups of patients receiving neoadjuvant chemotherapy based on 5 Fu or Gemcitabine to neoadjuvant chemoradiotherapy using 10 × 3 Gy or 28 × 1.8 Gy fractionation schedules in combination with a variety of drugs (Gemcitabine, capecitabine, DDP). The chemoradiotherapy group had a significantly higher R0 resection rate (91.2% vs. 79.2%), a lower rate of lymph node involvement (46.6% vs. 77.1%), and a lower incidence of locoregional recurrence (16% vs. 33%). No differences were observed in median survival time (33.6 m vs. 26.4 m) [15].

Takahasi et al. reported on the results of preoperative chemoradiotherapy in operable and BLRPC patients [16]. In a group of 80 patients with BLRPC, irradiation of the tumour, lymph node area of the visceral trunk, superior mesenteric and periaortic arteries was combined simultaneously with gemcitabine-based chemotherapy for cycle 1 and two subsequent cycles. In the group of patients with BLRPC, surgery was performed in 54% of patients. The reason for withdrawal of surgery was disease progression. The R0 resection rate was 98%. Locoregional relapses at 5 years was observed in 15% of operated patients. The percentage of patients surviving 5 years was 18% in the whole group and 34% in the operated patients [16].

In the PREOPANC-1 trial, patients with borderline resectable pancreatic cancer (BLRPC) were randomised to surgery within four weeks of randomisation with subsequent administration of six cycles of gemcitabine (a regimen similar to the CONKO-1 trial) or chemoradiotherapy (36 Gy in 15 fractions to the tumour area and suspicious lymph nodes) with concurrent, complementary gemcitabine and surgery within 14–18 weeks of randomisation. The R0 resection rate was 70% in the group of patients treated with neoadjuvant therapy compared to 40% treated initially with surgery. Similarly, lymph node metastases were found in a significantly lower proportion of patients in the neoadjuvant treatment group (33% vs. 78%). Approximately 10% of patients did not complete their planned chemoradiotherapy due to progression or toxicity. Approximately 12% of patients showed disease progression on CT scan after chemoradiotherapy. In the group of patients with BLRPC, the use of preoperative chemoradiotherapy was associated with significantly longer median survival (13.2 m vs. 17.6 m), time to locoregional recurrence (11.8% vs. 27.7%), and no significant effect on time to distant metastasis (12.2% vs. 21.5%) [17]. The mature results of the PREOPANC-1 trial presented after a median follow-up of 59 months indicated the persistence of a beneficial effect from neoadjuvant treatment in terms of significantly longer median survival time (14.3 m vs. 15.7 m) and survival rates of 3 and 5 years (16.6% vs. 27.7%, 6.5% vs. 20.5%) [18]. A statistically significant difference in survival time in favour of neoadjuvant treatment was observed in the BLRPC group [18].

In a 2021 analysis, Yoo Jin Choi et al. retrospectively compared the outcomes of neoadjuvant treatment with Gemcitabine and the FOLFIRINOX regimen. In the gemcitabine group, 79.5% of patients received radiotherapy, while in the FOLFIRINOX group, 28.8% of patients received radiotherapy. Survival outcomes as measured by 5yOS (46% vs. 19.1%) and distant metastasis rate (52.6% vs. 78.6%) were significantly better in the FOLFIRINOX chemotherapy group. The multidrug chemotherapy group also had a significantly higher proportion of patients experiencing serious side effects [19]. The use of the FOLFIRINOX chemotherapy regimen in BLRPC patients leads to resection in 68.5% of patients with R0 resection in 93% of these patients [20]. In the group of initially non-operable patients, these percentages are 22.5% and 86%, respectively [20].

Melion et al. presented a retrospective analysis including patients with BLRPC or locally advanced pancreatic cancer (LAPC). Of the 159 patients, two-thirds met criteria for BLRPC. Neoadjuvant treatment included a variety of systemic treatment regimens with subsequent stereotactic radiotherapy. A dose of 28–30 Gy was deposited in the tumour area with a margin of 3–5 mm with a simultaneous dose escalation to 50 Gy to the infiltrative area in the vascular region. Irradiation was delivered in 5 fractions. In the BLRPC group, resection was performed in 51% of patients, with R0 resection in 96% of them, while in the LAPC group, resection was performed in 10% patients, with R0 resection in all of them, respectively. Median OS and DFS were 19.2 m and 11.9 m for BLRPC, and 15 m and 13.2 m for LAPC, respectively. In patients with LAPC, the FOLFIRONOX regimen was associated with better treatment outcomes [21].

Neoadjuvant gemcitabine-containing chemoradiotherapy in BLRPC was compared to immediate surgery in a meta-analysis in 2022 [22]. FOLFIRINOX chemotherapy was not used after surgery in the analysed groups. The use of neoadjuvant chemoradiotherapy was associated with improved OS and higher R0 treatment rates [22].

In a phase II study, Murphy et al. presented a protocol for the management of 48 patients with BLRPC. After four cycles of chemotherapy according to FOLFIRINOX and assessment of the response in 44 patients, irradiation was applied to the tumour area with a 1 cm margin and elective lymph nodes (ENI): the celiac, porta hepatis, superior mesenteric, and paraaortic in a fractionation scheme of 5 × 5 Gy (proton radiotherapy) or 10 × 3 Gy (conventional radiotherapy). After an evaluation of the response, the patients were operated. Surgery was performed in 39 patients (81%). Tumour resection was abandoned in 7 patients due to disease progression, while resection was performed in 32 (66%) patients. R1 resection was performed in one patient (3%). Surgery was abandoned in 5 patients due to disease progression (2 patients), early death (2 patients), and potential toxicity (1 patient myocardial infarction) [23].

A retrospective analysis presented by Wang et al. suggests that the use of higher doses of radiotherapy in neoadjuvant treatment (54–64 Gy vs. 50.4 Gy) leads to higher resection rates among BLRPC patients [24].

In contrast to the optimistic treatment results presented above in the previous year, Hill et al. presented the results of 155 patients undergoing neoadjuvant chemotherapy (FOLFIRONOX or Gemcytabine/Nab-Paxlitaxel) and 33 Gy stereotactic radiotherapy in 5 fractions. Overall, 70 and 80% of patients in the BLRPC group and 63% in LAPC underwent tumour resection after neoadjuvant treatment, with R0 resection in 96% and 88% of them, respectively. Locoregional recurrence was observed in 33% of patients undergoing resection [23]. The median survival time from disease diagnosis was 26 months, while 1yOS and 2yOS were 93% and 51%, respectively [25].

A randomised phase 2 study, A021501 [26], yielded surprising results, with respect to the previously cited studies. One hundred and twenty-six patients with a diagnosis of BLRPC were assigned to receive eight cycles of mFOLFIRINOX vs. seven cycles of mFOLFIRINOX and stereotactic irradiation (33–40 Gy in 5 fr.) or image-guided radiotherapy (25 Gy in 5 fr.) before surgery. A total of 70 patients were treated with chemotherapy alone, while 56 were treated with irradiation. Postoperatively, four cycles of FOLFOX6 were administered in both arms. In the radiotherapy arm, R0 resection was performed significantly less frequently (57% vs. 33%). Due to the low resection rate in the 30 patients receiving combined treatment, further recruitment in this arm was discontinued. The median survival time in the radiotherapy group was significantly lower (17.1 m vs. 29.8 m) [26]. In the chemotherapy arm, treatment outcomes did not differ significantly from other studies reported previously [23,25]. Outcomes in the radiotherapy arm were significantly worse compared to other studies [23,25]. In the seven-cycle mFOLFIRINOX arm, grade 3 and higher toxicity (64% vs. 57%), deferral of subsequent treatment courses (60% vs. 49%), and reduction in cytostatic doses (75% vs. 60%) were observed more frequently during chemotherapy compared with the eight-cycle mFOLFIRINOX arm [26]. Operative treatment was performed in a similar proportion of patients, at 74% and 88%, respectively [26]. It is difficult to infer a lack of efficacy of radiotherapy based on the aforementioned data, other than to hypothesise that stereotactic radiotherapy cannot serve to compensate for the lower intensity of systemic treatment. The above-mentioned study also confirmed that the mFOLFIRINOX regimen delivers a significant improvement in treatment outcome, but at the price of a high proportion of patients experiencing side effects.

A summary of the data is presented in Table 1.

## 4. Treatment of Locally Advanced Pancreatic Cancer

The ECOG study compared the use of gemcitabine-based chemotherapy alone to combined concurrent chemoradiotherapy with gemcitabine in LAPC. In the experimental arm, 3D conformal radiotherapy was applied to the tumour area and ENI at a dose of 50.4 Gy fractionated conventionally. The use of radiotherapy resulted in a significant increase in median survival time from 9.2 months to 11.1 months, but at the cost of a significantly higher rate of grade 4/5 adverse events [27].

A retrospective analysis of the GERCOR group study compared the impact of the use of chemoradiotherapy after completion of three cycles of systemic treatment. A dose of 55 Gy in conventional fractionation was deposited to the tumour area and ENI. The use of radiotherapy was associated with significantly prolonged overall survival (median 15 months vs. 11.7 months), time to recurrence, and 1yOS (65.3% vs. 47.5%) [28].

In the randomised LAP07 trial, patients after four courses of gemcitabine-based systemic treatment were randomised to receive combination radiotherapy with capecitabine compared to continued gemcitabine-based systemic treatment. A dose of 54 Gy in classical fractionation was deposited to the tumour area and ENI with a margin of 1.5 cm. There were no statistically significant differences in survival time (median survival 15.2 months vs. 16.5 months) or grade 3/4 toxicity between the two arms. Local recurrence was observed less frequently in the irradiated group (32% vs. 46%), at the cost of a higher rate of distant metastases (60% vs. 44%) [29].

The FFCD/SFRO trial compared chemoradiotherapy (based on 5 Fu/DDP and irradiation to the tumour area and ENI to a dose of 60 Gy) with gemcitabine-based chemotherapy. Gemcitabine was followed in both arms until toxicity or progression. Worse treatment outcomes were observed in the chemoradiotherapy arm. Grade 3 and higher toxicity was more frequently observed in the chemoradiotherapy group. Overall, 75% of patients did not receive their planned dose of radiotherapy [30].

The SCALOP trial compared gemcitabine and capecitabine as components of combination treatment with chemoradiotherapy. Three cycles of systemic treatment (gemcitabine with capecitabine) were followed by combination treatment based on irradiation with a dose of 50.4 Gy at a conventional fractional dose deposited in the tumour area and enlarged lymph nodes with a margin of 1.5 cm. Seventy-four patients participated in the study. A statistically significant longer median OS (15.4 months vs. 13.2 months) in favour of capecitabine was observed, with lower grade 3/4 toxicity (0 vs. 18%) [31]. In a subsequent 2017 publication, no significant differences in median survival (17.6 months vs. 14.6 months) and time to progression (12 months vs. 10.4 months) were observed between the drugs used [32]. However, in a multivariate analysis, the use of capecitabine-based chemoradiotherapy was a predictor for longer survival time [32]. The use of chemoradiotherapy is associated with a significant reduction in quality of life; however, 3 weeks after the end of treatment, patient-reported quality of life returned to baseline [33].

The studies presented above used regimens of classically fractionated radiotherapy, with extensive margins around the tumour, using conformal radiotherapy techniques. Reported grade 3 and higher toxicity affected between 20% and 40% of patients [30,31,34,35]. Surrounding the pancreas are numerous organs and tissues sensitive to the application of ionising radiation such as the stomach, duodenal loop, large and small intestines, liver, kidneys, and spinal cord. Rakhra et al. compared two ways of delivering radiotherapy: hypofractionated irradiation (36 Gy in 15 fr.) of the tumour area with a 1 cm margin to classically fractionated irradiation (50.4 Gy in 28 fr.) to the tumour area with ENI and a 1 cm margin. Despite the use of a potentially more toxic drug in the hypofractionated radiotherapy arm (gemcitabine vs. capecitabine in the classically fractionated radiotherapy arm), better results were observed for 1yOS (57% vs. 36%) and lower late grade 3 and higher toxicity (11.9% vs. 19.2%) in favour of the hypofractionated regimen [36]. A dose escalation of 15% (to a dose of 57.25 Gy) to the tumour area using intensity-modulated radiotherapy (IMRT) techniques compared to a classically fractionated dose (50–50.4 Gy in 25–28 fractions) resulted in a longer median survival time (17.8 months vs. 15 months) and time to regional recurrence (10.2 months vs. 6.2 months) in patients undergoing chemoradiotherapy [37]. Grade 3 and higher toxicities were not observed in the mentioned study [37].

The main cause of treatment failure in patients with advanced pancreatic cancer are distant metastases, which were diagnosed in 50–70% of patients, while locoregional recurrence was diagnosed in approximately 40% [29,34,35]. The implementation of new drug regimens in the treatment of advanced pancreatic cancer has resulted, compared to gemcitabine in monotherapy, in prolonged survival times. For the mFOLFIRINOX regimen, median OS, 1yOS, and 2yOS are 23 m, 77.4%, and 46.2%, respectively, while for the combination of gemcitabine and nab-paclitaxel, they are 21.3 months, 82.5%, and 41.3%, respectively [38]. Grade 3 and higher toxicity affects approximately one-quarter of patients treated with those regimens [39].

The development of irradiation methods in the form of a shift from conformal radiotherapy techniques to IMRT has contributed to a significant reduction in the proportion of patients experiencing early grade 3 reactions (i.e., vomiting, diarrhoea) from 11–18% to 3–7.8% [40,41]. With further advances in technology, it has become possible to image the tumour taking into account its mobility due to respiration. Performing a CT scan taking into account the phases of breathing allows the position of the tumour to be reconstructed during the different phases of breathing. Based on the images acquired in this way, it is possible to plan and execute irradiation at selected respiratory phases. Simultaneous imaging techniques available on linear accelerators allow the position of the tumour to be reproduced with high precision, taking into account the selected respiratory phases. All the above-mentioned factors have made it possible to implement stereotactic radiotherapy in the treatment of pancreatic cancer. With the reduction in irradiated healthy tissues volume (restriction of the irradiation volume to the tumour instead of ENI; use of narrow margins around the tumour of 0–5 mm), it is possible to deposit much higher doses in the tumour without a significant increase in the risk of side effects.

The available publications describing the use of stereotactic radiotherapy in patients with advanced pancreatic cancer concern relatively small groups of patients (30–75) and are retrospective in nature [42,43,44,45,46]. The use of stereotactic radiotherapy occurred after systemic treatment, mostly based on gemcitabine. The area of irradiation included the pancreatic tumour and possibly enlarged lymph nodes with a margin of 3–5 mm [42,43,44,45,46]. The dose prescribed for the tumour was 33–35 Gy in 5 fractions [42,43,44] or 45 Gy in 6 fractions [45,46]. In the groups of patients irradiated with the higher dose, 1y local control (LC) of 86% was achieved compared to 78% for the lower doses [43,44,45,46]. 1yPFS was 32–41% [42,44,46] while 1yOS was 47–85% [42,44,45,46]. One has to interpret the mentioned data with great caution due to the retrospective nature, the different treatment eligibility tools and defining from when PFS and OS times were calculated. For example, three studies did not clearly define how to calculate follow-up times [42,43,45], one calculated from the time of diagnosis [44], and another from the date of completion of irradiation [46]. One study clearly indicated that PET/CT was performed before treatment in 98% of patients [44]. The toxicity and analgesic effect data are important information from these studies. Early grade 3 and higher toxicity was observed in about 10% of patients [44], while late toxicity was observed in 0 to 5.3% [42,44,45,46,47]. Approximately 50% of patients discontinued analgesics after irradiation, while approximately 25% reduced analgesic doses to 50% of the original level [45,46]. Unfortunately, one study observed a recurrence of pain to the originally reported level after a period of approximately 4 months [43]. In an analysis of irradiation schedules for patients undergoing radiotherapy at a dose of 25 Gy in 5 fr. to the tumour area, it was shown that in two-thirds of cases, it is possible to increase the dose to at least 40 Gy in an area of perivascular infiltration without increasing the risk of significant side effects [48].

There are no studies directly comparing the use of stereotactic radiotherapy to conventional radiotherapy. Retrospective analyses are available comparing treatment outcomes with both irradiation modalities. A retrospective analysis of data from the NCDB shows a benefit of stereotactic radiotherapy in terms of a significant increase in the 2yOS from 16.5% to 21.7% [49]. In the cited study, the discriminating factor between the two radiotherapy techniques was only the fractional dose: for stereotactic radiotherapy, it was ≥4 Gy, while conventional radiotherapy was ≤2 Gy. It is also important to note that the proportion of patients with T4 and N+ features was higher in the conformal radiotherapy group [49]. In contrast, in a meta-analysis of studies using stereotactic and conventional radiotherapy, the authors defined the two groups differently: stereotactic radiotherapy at a fractional dose ≥ 5 Gy and number of fractions ≤ 5 (most 30 Gy in 5 fr.), with conventional radiotherapy at a fractional dose of 1.8–2 Gy (most regimens 45–50.4 Gy, concurrent chemoradiotherapy) [50]. The proportion of patients surviving 2 years was significantly higher in the stereotactic irradiation group (26.9% vs. 17.3%) [50]. Grade 3 early toxicity and higher was less frequently observed in the stereotactic irradiation group (5.6% vs. 37.7%) [50]. In terms of late toxicity, the two irradiation methods did not differ [50].

The significant difference in early toxicity is due to the smaller volume of healthy tissue in the area of the relevant ionising radiation dose in favour of stereotactic radiotherapy. Wild et al. compared groups of patients treated with stereotactic and conventionally fractionated radiotherapy [48]. In the stereotactic radiotherapy group, a dose of 33 Gy in 5 fr. was deposited on the tumour area with a margin of 3–5 mm, while in the conventionally fractionated radiotherapy group, a dose of 50.4 Gy in 28 fractions was deposited on the tumour area with ENI and a margin of 1.5–2.5 mm. Systemic treatment was administered in both groups. The median volume of the planned irradiation area was significantly smaller in the stereotactic radiotherapy group (88.7 cm^3^ vs. 334.6 cm^3^) [51]. A smaller tissue area, including vessels, led to a lower incidence of severe lymphopenia (<500 cells/mm^3^) in the stereotactic radiotherapy group at 1 and 2 months after the start of radiotherapy, respectively (13.8% vs. 71.1% and 13.6% vs. 46%) [49]. Higher values of lymphocyte levels were associated with longer survival regardless of treatment regimen [51].

The CT scan, performed for treatment planning purposes, reflects the anatomical situation and the position of the tumour in relation to the stomach, duodenum, intestines, kidneys, and liver. Due to the mobility of these organs associated with respiration, peristalsis and the variable filling of food and its residues on each day of irradiation, the anatomical situation varies. In some situations, this may result in an important part of the healthy tissues being in the planned high-dose area. The combination of a linear accelerator and magnetic resonance-based imaging (MRlinac) in a single machine facilitates the use of stereotactic MR-guided adaptive radiotherapy (SMART) procedures. Prior to each irradiation session, MR imaging is performed in the therapeutic position. Based on the acquired images, a plan is created, taking into account the primary plan, which takes into account the anatomical conditions present at the time. Data presented by Bordeau et al. indicate that significant adaptation was realised for each irradiation fraction [52]. The authors presented the results of treating 70 patients using SMART. A dose of 50 Gy in 5 fr. was deposited to the tumour area with a margin of 3 mm. Overall, 87% of patients received chemotherapy before irradiation. The group presented was heterogeneous. For example, there were 63 patients in the study group with a diagnosis of pancreatic adenocarcinoma, including 52 with BLRPC and LAPC. In six patients, the pancreatic lesions were secondary. Local recurrence was observed in three (8.6%) patients, including one in the tumour and two at the tumour margin [52]. No grade 3 or higher toxicity was observed, while 1yOS and 2yOS were 91% and 45.8%, respectively [52]. Another retrospective analysis, presented by Chuong et al., presents the results of treatment with SMART in a group of 62 patients diagnosed with advanced pancreatic cancer, including 14 with BLRPC [53]. More than 90% of these patients initially received chemotherapy based on FOLFIRINOX or gemcitabine and nab-paclitaxel regimens. In the group presented here, the irradiation area was defined differently. A dose of 33 Gy in 5 fr. was deposited over the tumour area and celiac trunk and superior mesenteric vessels with a 3 mm margin, while a dose of 50 Gy was deposited over the tumour area with a 3 mm margin. The 1yLC and 2yLC results presented were 98.3% and 87.7%, respectively [53]. Median OS results of 23 m, 1yOS 90.2%, and 2yOS 45.5%, were similar to studies with modern systemic treatment regimens [35,36,50]. 1yPFS and 2yPFS were 88.4% and 40%, respectively [53]. Early and late grade 3 and higher toxicities were observed in 4.8% of patients [53]. Analysis of local recurrences showed that one-third of these were localised within the tumour and two-thirds outside the tumour [54]. Limitations for the use of SMART are the high cost of the equipment required for this technique, and the relatively long lead time for the procedure. The implementation of real-time adaptive radiotherapy using commonly used linear accelerators may contribute to a significant uptake of this treatment modality.

Parisi et al. presented the COMBO-Therapy concept to combine benefits of concomitant chemoradiotherapy and stereotactic radiotherapy [55]. A group of 13 patients with LAPC received induction chemotherapy in the form of 4–6 months of chemotherapy with gemcitabine in monotherapy or in combination with nap-paclitaxel followed by concurrent chemoradiotherapy. A dose of 50.4–54 Gy at a fractional dose of 1.8 Gy per tumour area and ENI was given in combination with capecitabine. Eight patients subsequently received stereotactic irradiation to the tumour area at a dose of 10–21 Gy in 1–3 fractions. In the group of five patients who did not receive SRT, three experienced disease progression, one cardiovascular complications, and one patient underwent surgery. The 2yLC and 2yOS of the eight patients who completed treatment were 72.9% and 53.9%, respectively [55].

A summary of the data is presented in Table 2.

Studies using stereotactic radiotherapy and SMART show that it is possible to safely deposit high doses of irradiation to achieve high local cure rates. Patient volumes in publications using stereotactic techniques indicate that this method applies to selected groups of patients. In many clinical cases, conventionally fractionated or hypofractionated regimens (d.fr. = 2.4 Gy) combined with concurrent chemotherapy may still be more beneficial. There is no apparent evidence to support the use of ENI. The optimal place to use radiotherapy in advanced pancreatic cancer is after systemic treatment and confirmation of no disease progression. A high proportion of patients experience a significant reduction in pain intensity after irradiation, and it therefore seems reasonable to also consider the use of appropriately planned and administered radiotherapy in selected cases of patients with generalised cancer.

## 5. Conclusions

Generalisation of the tumour process remains the most common cause of treatment failure in pancreatic cancer.

The implementation of new systemic treatment regimens contributes to improved treatment outcomes irrespective of the stage of the disease. To improve treatment outcomes in terms of the incidence of distant metastases, the impact of local curability on the length and quality of life of patients increases.

Modern radiotherapy achieves high local cure rates.

Postoperative radiotherapy in combination with chemotherapy seems justified in a group of postoperative pancreatic cancer patients with pT3 and pN+ features.

In patients with borderline resectable pancreatic cancer, the impact of radiotherapy in combination with the latest chemotherapy regimens is difficult to define clearly.

If advanced pancreatic cancer is diagnosed, radiotherapy, especially stereotactic radiotherapy, in combination with chemotherapy, contributes to an improved local cure rate and significantly reduces the level of pain.

## Figures and Tables

**Table 1:** Studies including neoadjuvant radiotherapy in pancreatic cancer.

	Study Protocol	Radiotherapy Protocol	R0 ^1^	pLN+ ^2^	Results	Toxicity Grade ≥ 3
Takahashi [16]	Gem ^3^ + RT ^4^ + S ^5^ Operable (188p) vs. BLRPC ^6^ (80p)	3dCRT ^7^: GTV ^8^ + ENI ^9^, 25 × 2 Gy	87% vs. 52%		LRR ^10^ 13% vs. 15% 5yOS ^11^ 54% vs. 18%	H ^12^ 48.3% GI ^13^ 3%
Cloyd [15]	Gem or 5 Fu + S (37p) vs. Gem/Cap ^14^/DDP ^15^/RT + S (227p)	3DCRT: GTV + 10 mm + ENI, 10 × 3 Gy or 28 × 1.8 Gy	79.2% vs. 91.2%	77.1% vs. 46.6%	LRR 33% vs. 16% median OS 26.4 m vs. 33.6 m	
PREOPANC1 [18]	BLRPC S + 6xGem (120) vs. 3xGEM + 3xGem/RT + S + 4xGem (128)	GTV + ILN ^16^: 15 × 2.4 Gy	40% vs. 70%	78% vs. 33%	LRR RR: 0.57 median OS 14.3 m vs. 15.7 m 3yOS 16.6% vs. 27.7%	
Yoo Jin Choi [19]	BLRPC Gem + S (34) vs. FOLFIRINOX + S (66)	79.5% RT in Gem arm vs. 28.8% RT in FOLFIRINOX arm	94.1% vs. 92.4%	29.4% vs. 40.9%	2yDFS ^17^ 29.4% vs. 45.1% median OS 27 m vs. 28 m 2yOS 58.4% vs. 72.2% 5yOS 19.1% vs. 46%	2.9% vs. 21.2%
Melion [21]	BLRPC Gem or FOLFIRINOX or NabPXL ^18^/GEM + RT + S (110)	IMRT ^19^: GTV + 3 mm, 5 × 4.6–5 Gy, CT ^20^/AMS ^21^ 5 × 8–10 Gy	49.1%	41%	median OS 19.2 m	3.7%
	LAPC Gem or FOLFIRINOX or NabPXL/GEM + RT + S (49)		10.2%	60%	median OS 15 m	
Murphy [23]	BLRPC 8xFOLFIRINOX + Cap/RT + S (48p)	GTV + 1 cm + ENI, Protons 5 × 5 Gy 10 × 3 or 28 × 1.8 Gy	65%		LRR 6% 2yPFS ^22^ 43% median OS 37.7 m 2yOS 56%	19%
Hill [25]	BLRP/LAPC ^23^ FOLFIRINOX or NabPXL/GEM + RT + S (155)	SBRT ^24^: GTV + 2 mm, 5 × 6.6 Gy	63.2%	41%	LRR 33% median OS 26 m 2yOS 51%	
A021501 [26]	BLRPC 8xFOLFIRINOX + S + 4xFOLFOX6 (70p) vs. 7xFOLFIRINOX + RT + S + 4xFOLFOX6 (56p)	SBRT: GTV +3 mm, 5 × 6.6–8 Gy IGRT ^25^: GTV + 5–10 mm, 5 × 5 Gy	57% vs. 33%	47% vs. 47%	median OS 29.8 m vs. 17.1 m 18 mOS 66.7% vs. 47.3%	57% vs. 64%

^1^ R0—percentage of patients who underwent radical resection; ^2^ pN+—percentage of patients with metastases in lymph nodes in pathological examination; ^3^ Gem—gemcitabine-based chemotherapy; ^4^ RT—radiotherapy; ^5^ S—surgical treatment; ^6^ BLRPC—borderline resectable pancreatic cancer; ^7^ 3dCRT— three-dimensional conformal radiotherapy; ^8^ GTV—gross tumour volume; ^9^ ENI—elective nodal irradiation; ^10^ LRR—locoregional relapse rate; ^11^ OS—overall survival; ^12^ H—haematological toxicity; ^13^ GI—gastrointestinal toxicity; ^14^ Cap—capecitabine; ^15^ DDP—cisplatin; ^16^ ILN—involved lymph nodes; ^17^ DFS—disease-free survival; ^18^ NabPXL—nab paclitaxel; ^19^ IMRT—intensity-modulated radiotherapy; ^20^ CT—celiac trunk vessels; ^21^ AMS—mesenteric superior artery; ^22^ PFS—progression-free survival; ^23^ LAPC—locally advanced pancreatic cancer; ^24^ SBRT—stereotactic body radiotherapy; ^25^ IMRT—image-guided radiotherapy.

**Table 2:** Studies including radiotherapy in locally advanced pancreatic cancer.

	Study Protocol	Radiotherapy Protocol	Results	Toxicity Grade ≥ 3
ECOG [27]	Gem ^1^ (37) vs. Gem/RT ^2^ (34)	3dCRT ^3^: GTV ^4^ + 3 cm, ENI ^5^ 22 × 1.8 Gy, GTV + 2 cm 6 × 1.8 Gy	median OS ^6^ 9.2 m vs. 11.1 m	77% vs. 79% Grade ≥ 4.9% vs. 4.1%
Huget [28]	Gem (56) vs. Gem + 5 Fu/RT (72)	3dCRT (SIB): GTV + ENI 25 × 1.8 Gy, GTV + CT ^7^/AMS ^8^ 8 × 1.25 Gy	median PFS ^9^ 7.4 m vs. 10.8 m median OS 11.7 m vs. 15 m 1yOS 47.5% vs. 65.3%	
LAP07 [29]	Gem (136) vs. Gem + Cap ^10^/RT (133)	GTV + 15 mm/30 mm 30 × 1.8 Gy	LRR ^11^ 46% vs. 32% DMR ^12^ 44% vs. 60% median OS 15.2 m vs. 16.5 m	H ^13^ 10.4% vs. 3.9% GI ^14^ 19.8% vs. 23.1%
FFCD/SFRO [30]	Gem (60) vs. 5 Fu/DDP ^15^/RT + Gem (59)	3dCRT: GTV + ENI 30 × 2 Gy	1yPFS 32% vs. 14% median OS 13 m vs. 8.6 m 1yOS 53% vs. 32%	H 27.3% vs. 30.9% GI 18.2% vs. 43.6%
SCALOP [31]	Gem/Cap + Cap/RT (36) vs. Gem/Cap + Gam/RT (38)	3dCRT/IMRT: GTV + 5 mm/2 mm 28 × 1.8 Gy	median PFS 12 m vs. 10.4 m median OS 17.6 m vs. 14.6 m	12% vs. 37% H 0 vs. 18% GI 0 vs. 16%
Rakhra [36]	5 Fu/RT vs. Gem/RT	3dCRT: 5 Fu: GTV + ENI + 1 cm 28 × 1.8 Gy Gemcitabine: GTV + 1 cm 15 × 2.4 Gy	1yOS 36% vs. 57% 2yOS 6% vs. 36%	GI 19% vs. 12%
Chuong [43]	3xGem + RT (64)	SBRT ^16^: GTV + 3–5 mm 5 × 6 Gy CT/AMS 5 × 7–10 Gy	1yPFS 41% median OS 15 m 1yOS 68.1%	Acute 0 Late 5.3%
Herman [44]	3xGem + RT + Gem (49)	SBRT: GTV + ILN ^17^ 5 × 6.6 Gy	1yLRR 22% median PFS 7.8 m 1yPFS 32% 2yPFS 10% median OS 13.9 m 1yOS 59% 2yOS 18%	Acute: GI 2% H 8.2% Late GI: 8.5%
Tozzi [45]	Gem + RT (30)	SBRT: GTV + 5/10 mm 6c7.5 Gy	1y LRR 23% median OS 11 m 1yOS 47%	Acute 0
Rwigema [47]	Chemo ^18^ + RT (71)	SBRT: GTV 18–25 Gy/1–4 fractions	1yLRR 51.5% median OS 10.3 m 1yOS 41%	Acute 4.2% Late 0
Bordeau [52]	FOLFIRINOX (50%) or Gem (4%) + RT (70)	SMART ^19^: GTV 5 × 10 Gy	1yLRR 13.5% 2yLRR 19.3% median OS 20.9 m 1yOS 68.6% 2yOS 37.7%	Acute 0 GI Late 2%
Chuong [54]	Chemo (69.4%) + RT (62)	SMART: GTV 5 × 10 Gy	1yLRR 1.7% 2yLRR 12.3% median OS 23 m 1yOS 90.2% 2yOS 45.5%	Acute 4.8% Late 4.8%
Parsi [55]	4xGem/Nab-PXL + Cap/RT + SBRT (13)	IMRT: GTV + ENI 25 × 2 Gy SBRT: GTV 10–21 Gy/1–3 fractions	2yLRR 17.1% median OS 21.5 m 2yOS 53.9%	No G3

^1^ Gem—gemcitabine-based chemotherapy; ^2^ RT—radiotherapy; ^3^ 3dCRT—three-dimensional conformal radiotherapy; ^4^ GTV—gross tumour volume; ^5^ ENI—elective nodal irradiation; ^6^ OS—overall survival; ^7^ CT—celiac trunk vessels; ^8^ AMS—mesenteric superior artery; ^9^ PFS—progression-free survival; ^10^ Cap—capecitabine; ^11^ LRR—locoregional relapse rate; ^12^ DMR—distant metastasis rate; ^13^ H—haematological toxicity; ^14^ GI—gastrointestinal toxicity; ^15^ DDP—cisplatin; ^16^ SBRT—stereotactic body radiotherapy; ^17^ ILN—involved lymph nodes; ^18^ Chemo—chemotherapy; ^19^ SMART—stereotactic MR-guided adaptive radiotherapy.

## Data Availability

The research led to creation of no data to share.
